# Structure Based Antibody-Like Peptidomimetics

**DOI:** 10.3390/ph5020209

**Published:** 2012-02-16

**Authors:** Ramachandran Murali, Mark I. Greene

**Affiliations:** 1 Department of Biomedical Sciences, Cedars-Sinai Medical Center, D5091 Davis Building, 8700 Beverly Blvd., Los Angeles, CA 90048, USA; 2 Department of Pathology and Laboratory of Medicine, Perelman School of Medicine, University of Pennsylvania, Philadelphia, PA 19104, USA

**Keywords:** antibody, CDR, peptidomimetics, Her2, Herceptin, drug-delivery, therapeutics, tumor imaging, AHNP, AERP

## Abstract

Biologics such as monoclonal antibodies (mAb) and soluble receptors represent new classes of therapeutic agents for treatment of several diseases. High affinity and high specificity biologics can be utilized for variety of clinical purposes. Monoclonal antibodies have been used as diagnostic agents when coupled with radionuclide, immune modulatory agents or in the treatment of cancers. Among other limitations of using large molecules for therapy the actual cost of biologics has become an issue. There is an effort among chemists and biologists to reduce the size of biologics which includes monoclonal antibodies and receptors without a reduction of biological efficacy. Single chain antibody, camel antibodies, Fv fragments are examples of this type of deconstructive process. Small high-affinity peptides have been identified using phage screening. Our laboratory used a structure-based approach to develop small-size peptidomimetics from the three-dimensional structure of proteins with immunoglobulin folds as exemplified by CD4 and antibodies. Peptides derived either from the receptor or their cognate ligand mimics the functions of the parental macromolecule. These constrained peptides not only provide a platform for developing small molecule drugs, but also provide insight into the atomic features of protein-protein interactions. A general overview of the reduction of monoclonal antibodies to small exocyclic peptide and its prospects as a useful diagnostic and as a drug in the treatment of cancer are discussed.

## 1. Introduction

Recent advances in gene expression, protein production and protein engineering have led to the realized use of macromolecules as therapeutic agents. Antibodies represent a powerful class of therapeutics useful to treat various pathologies [1,2]. There is a need for smaller size of molecular agents, easier to deliver (*i.e*., orally) and less expensive to produce. Reducing a macromolecule into a smaller molecule or finding a small molecule to alter the function of target protein is still a challenge. Conventionally, small molecules are discovered either from a routine high throughput screening or some other equivalent screening methods. Currently there are no facile structure based routine methodologies available to convert a macromolecule suitable for therapeutic use into a deconstructed organic structure although our laboratory and Kahn’s group have described a general synthetic approach to create loop mimics of cyclic subunits of proteins [3]. Alternative avenues to antibodies are creating a mini-proteins [4,5,6] and Fc-fused proteins [7,8,9].

Human insulin was the first protein that was successfully produced using DNA technology for the treatment of diabetics [1,10,11]. Since then several recombinant proteins have been introduced for clinical use [12,13,14,15,16]. There are currently at least about 350 proteins being developed by biotechnology companies [17] and over 30% of them belong to the class of antibodies. Advances come from the understanding of several features such as protein’s affinity, half-life and immunogenicity. Technology of recombinant protein production has also improved [17]. However, the major impediments in using proteins as drugs remain and are their poor tissue penetration, inability to cross blood-brain barrier and complex pharmacokinetics, toxicity and drug delivery [18,19,20,21,22].

Several therapeutic monoclonal antibodies (mAbs) have been approved or are in clinical trial in one or more major markets. Furthermore several radiolabeled mAbs have been approved or are being evaluated for *in vivo* imaging [2,23,24,25,26]. Some difficulties that have had to be overcome in recombinant antibody therapeutic application relate to immunogenicity [27]. The conventional route to derive mAbs is to immunize mice with antigen or peptide fragments derived from the antigen. Such murine mAbs have widespread applications in research, but can trigger immune responses because of the foreign nature of the protein when introduced into humans. Several approaches have been taken in overcoming this problem, which has seen the development of chimeric, humanized and now fully human mAbs [28,29,30].

Reducing a large size protein into a smaller molecule or creating a small molecule peptide mimic of the parent protein is an active area of research pursued by several laboratories [4,31,32,33,34,35]. The central philosophy in creating a mini-protein is to identify small structural domains or a scaffold and engineer it for high affinity, specificity and immunogenicity. For example, removal of a natural domain in tissue plasminogen activator (tPA) was enough to enhance its usefulness as a therapeutic agent for myocardial infarction [17].

Small molecular mimics are often designed by using a random screen such as phage display [35,36,37,38,39]. In contrast to random screens we have developed a rational structure based strategy to design peptidomimetics from proteins, receptors and immunoglobulins [40,41,42,43,44,45,46,47,48]. Here we focus on design of peptidomimetics from monoclonal antibody with more emphasis on anti-erbB peptidomimetics (AHNP, AERP) designed from the monoclonal antibody trastuzumab (Herceptin^®^, Genentech, Inc.) and anti-EGFR antibodies, respectively [48,49]. The review is divided into three sections; (1) overview of the structure of antibody which is the basis for much of the progress today, (2) then a brief overview of antibodies engineered for clinical use and their limitations and (3) finally the design and development of anti-erbB peptidomimetics.

## 2. Structure of Immunoglobulin

Successful use of monoclonal antibody in clinical use comes from our understanding of the structure of antibody. This section gives a brief overview of the antibody structure for the readers who are unfamiliar with the structural aspects of antibody.

**Figure 1:**
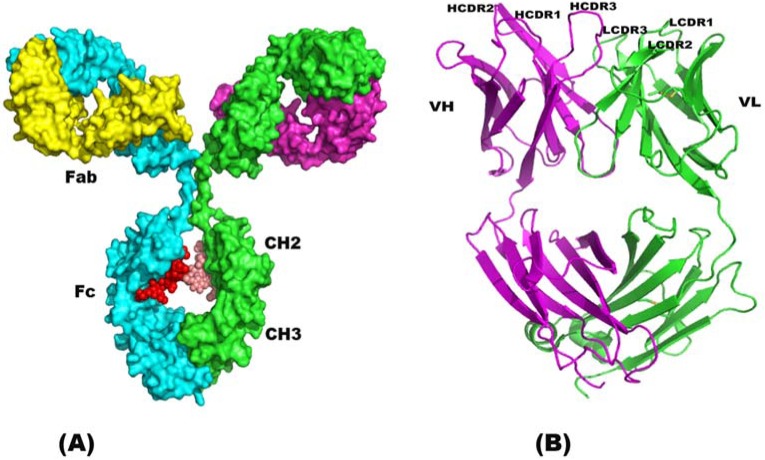
(**A**) Three-dimensional structure of antibody structure (protein data bank code: Igg1.ent). Antibody is a Y-shaped molecule with two arms (Fabs) and a stem (Fc region). These two domains are connected by disulfide links. The linkers allow a flexible movement in the antibody. Carbohydrates in the Fc region are shown as small red and pink spheres. (**B**) Antigen binding domain, Fab is shown in ribbon representation. Light and heavy chains are shown in green and purple, respectively. Fab domain is characterized by β-strands sandwiched as shown and interleaved with loops called complementary determining region (CDR). Six CDR loops mediate antigen specificity and binding. Pictures were created using Pymol [54].

Antibodies are composed of two polypeptide chains called “Light chain” and “Heavy chain” and often denoted by “L” and “H” respectively. The general structure is shown in Figure 1. Each light chain consists of variable domain (VL) and one constant domain (CL); and each H chains consist of one of the VL and three constant domains (C_H_1, C_H_2 and C_H_3) (Figure 1). Each domain exhibits a characteristic topology called the “immunoglobulin” domain. The three dimensional structure of the immunoglobulin domain consists of anti-parallel β-sheets arranged in a “sandwich” fashion (Figure 1). Structurally the variable and the constant domains are similar, except the variable domain possesses an extra pair of β-sheet strand and an extra loop connecting them. The two sides of the sandwich motif is covalently linked by disulfide bonds. Variable forms of the immunoglobulin fold have been widely identified in immune modulators, and viral receptors [50,51,52,53].

Antibody topology can be further divided into two parts: (1) Framework and (2) antigen binding regions (complementary determining region). The sequence variability in the VL and VH are limited to certain regions called “hypervariable regions” which forms the antigen binding site of the molecule, and are also called “complementarity determining region (CDR)”. The remainder is referred to as the framework region. The dispositions of the CDRs with respect to framework are shown in Figure 1. Each CDR is a loop connecting two β-strands, and has a fixed orientation on the framework, depending on its length and sequence characteristics of the individual domain. The VL and VH domains associate non-covalently to form a β-barrel structure. This association brings the three CDRs from VL and VH together at the binding site. Thus the binding site composed of 3 CDRs from VL and 3 CDRs from VH, six CDRs determines the specificity and affinity of antigen binding.

Our current knowledge on how antibody-antigen interactions occur at the atomic level comes from several crystallographic structures of Fab-antigen complexes [55,56]. It is now clear that the six CDR loops determine the antigen binding in terms of specificity and affinity. Chothia and Lesk [57] compared the conformation of CDR loops from several Fab crystal structures, and identified a common fold in CDR1 and CDR2 of light and heavy chains, and termed the standard fold (*i.e*., polypeptide main chain conformation) as “canonical structures”. The canonical structures adopted by CDR1 and CDR2 loops are determined by a few key residues, but independent of amino acid composition of hypervariable or CDR loops. However, in general, the CDR3 loops from VL and VH do not follow this rule, and are known to adopt “non-canonical” conformations.

CDR3 regions in an antibody play a critical role in the antigen binding and recognition. Analysis of several antigen-antibody complex from crystallography revealed that CDR3 from heavy chain makes most of the contacts with the antigen [56,58,59]. Further analysis of CDR loops and binding site reveal an imprecise correlation between the size of the antigen, number of contacts and affinity [60]. Interestingly, naturally occurring camelid antibodies lack light chains and contain only heavy chains. The heavy chains possess extended and long CDR3 loops which mediate high specificity and affinity [61,62]. The camelid observations of binding by a single CDR coupled with our creation of isolated CDR mimetics answer the question of whether specificity and affinity can be achieved by engineering CDR loops alone.

The independent functional role played by framework and CDR in antigen binding led to the development of three different engineered products; (1) Single chain antibody variable domain (Fv), which is a combination of VL and VH linked by a flexible linker (2) Chimeric antibody and (3) humanized monoclonal antibody by CDR grafting [63]. We believe that our demonstration that CDR loops function in a context independent manner predicted the success of CDR grafting.

### 2.1. Single Chain Antibody

Single-chain Fvs (scFvs) are recombinant antibody fragments consisting of only the variable light chain (VL) and variable heavy chain (VH) domains covalently connected to one another by a flexible polypeptide linker. The length of the linkers plays a role in the oligomerization of Fvs [64,65,66]. Single-chain Fvs also show a concentration-dependent tendency to oligomerize [64,67,68]. Bivalent scFvs are formed when the variable domains of a scFv disassociate from one another and are induced to reassociate with the variable domains of a second scFv. Similar rearrangement and reassociation of variable domains from different sFvs can result in the formation of trimers or higher multimeric oligomers [68]. Each Fv in a bivalent or multivalent Fv is composed of the VL domain from one scFv and the VH domain from a second sFv. Modifying the linker length or the inclusion of antigen may stabilize the VL/VH interface against rearrangement such that specific multimeric or monomeric forms of scFvs may be isolated. Structural studies from Nuclear magnetic resonance (NMR) and X-ray crystallography show that the scFv linker is highly flexible and disordered suggesting that the peptide may adopt a random coil-like structure [68,69]. Comparison of CDR loops in Fab and scFv show that the conformation remains same [70] suggesting that CDR loops remain independent and their conformation is critical for antigen recognition. Smaller size scFvs have an advantage over monoclonal antibody in terms of rapid pharmacokinetics and tumor penetration *in vivo* [71,72,73].

Single chain antibodies (ScFv) are being developed as candidates for drug-delivery and tumor imaging [74,75]. Furthermore, scFv are useful for creating bispecific antibodies [76,77,78]. Despite the promise of great utility for medical applications, scFv usage has been disappointing [79] reflecting the fact that improved affinity and size-reduction of this type of molecule are still inadequate or not optimally understood in a way sufficient for their use in clinics.

### 2.2. Humanization of Monoclonal Antibody

Monoclonal antibodies have proven to be useful drug in treating several diseases ranging from autoimmune processes, cancer and other pathologies [2,14,80,81,82,83]. The specificity of mAb and ease with which they can now be produced using recombinant DNA technology have made them viable therapeutic agents. Two major difficulties in using xenogenic (mostly murine) antibodies have been identified: (1) xenogenic antibodies do not always trigger the appropriate human effector systems of complement and Fc receptors [28,84], and (2) xenogenic antibodies can be recognized by a human anti-murine-antibody immune response (HAMA) and cleared quickly reducing the *in vivo* efficacy [85,86]. Atomic level structural understanding has made it easier to overcome some of the major drawbacks such as immunogenicity and led to molecular ways to increase half-life [14,17].

There are two distinct methods used in creating a humanized antibody; (1) CDR grafting [63] and (2) resurfacing (*i.e*., modifying surface residues to match human form of antibody framework regions) [87]. In the first approach, the human antibody framework is retained but CDR loops are spliced from their murine origin [88,89]. Detailed description of these two procedures is beyond the scope of this review and will be not discussed further. Winter *et al.* [90] reviewed the details of humanization by CDR grafting. Resurfacing is also discussed in other reviews [27,87,91,92].

### 2.3. Proteins to Peptides

Though humanized antibodies are used in clinical settings, humanizing a xenogenic antibodies does not preclude its safety, and still it can elicit anti-idiotypic and anti-allotypic responses after repeated administration [27]. Optimally humanized antibodies which induce limited anti idiotypic reactions possess relatively long circulating half lives [93].

Macromolecules such as full-length humanized mAbs still possess disadvantageous characteristics for clinical application: These problems include (1) commercial-scale production may be either difficult or costly, (2) macromolecules may be excluded from compartments such as the blood/brain barrier, (3) macromolecules have limited penetration into tissues [18,94] and (4) macromolecules including mAbs may induce severe side effects such as induction of anti-idiotypic antibodies and immune complex formation [27,28].

Smaller peptides represent obvious alternatives to mimic larger macromolecular structures when only a defined surface of the protein mediates activity. Design of peptide mimetics has been based on both structural and functional data [41,95,96,97,98]. While structure-based mimetic derivations can often mimic the parent protein functionally, generally these mimetics have less biologically active potency. Peptides identified from phage display are occasionally highly potent [99,100], but may not structurally resemble or mimic multiple functions of the parent protein. For example, the erythropoietin (EPO) mimetic identified from phage display do not have any homology with EPO, but able to mimic the hormone function [100]. In recent years, there has been a significant progress in the field of peptide chemistry and now peptide mimetics can be used as a template, and through iterations that reduce size and increase biological activity, may lead to viable therapeutic reagents [3,41,42,43,48,96,101,102,103,104,105,106,107,108,109].

### 2.4. Mimicry of Antigen by Peptides

Synthetic peptides can mimic the structural and biological characteristics of native proteins [110,111,112,113]. Synthetic peptides derived from CDR regions of monoclonal antibodies have been shown to act as a surrogate idiotype (Id) of the antibodies when used as immunogens [84,114,115,116]. Also, conformations of peptides co-crystallized with mAb have been shown to closely resemble the conformations of their cognate sequences in the native proteins [117,118], and many synthetic peptides derived from native proteins have been shown to biologically mimic those proteins [119,120,121]. The determinants that have been studied in detail do not have to be derived from a contiguous primary sequence in the native antigen to be represented by peptides, but instead can be conformational structures comprised of non-continuous residues brought together by folding. For example, a hexapeptide that block antibody induced myasthenia gravis-like symptoms in chicken obtained from a phage display library was shown to mimic a conformational epitope displayed on the acetylcholine receptor, but has the amino acid composition entirely different from the acetylcholine receptor [122].

Occasionally, an internal image anti idiotypic antibody (Ab2) will share sequence homology with the relevant homologous antigen. Of course, sequence homology is not always found in structurally related molecules. Lescar *et al*. [59] and Malby *et al*. [123] provide a clear example of two Fabs having similar specificity, yet the antigen binding sites are comprised of dissimilar sequences.

Synthetic peptides corresponding to areas of primary sequence shared between antibodies and the appropriate protein homologues duplicate functional idiotopes (Id) [124,125,126,127,128,129,130]. For example, PAC1, an anti-platelet fibrinogen receptor (GPIIβ/IIIα) antibody, has a RYD sequence in its unique third CDR of the heavy chain (H3) which is homologous to RGD of fibrinogen. A synthetic peptide encompassing the H3 region inhibited fibrinogen dependent platelet aggregation, as well as PAC1 and fibrinogen binding to activated platelets [128]. Molecular modeling studies of the H3 site indicate that it occupies the same conformational space as the RGD site in bioactive GPIIβ/IIIα [131]. Two anti-thyroid stimulating hormone (TSH) receptor mAbs, 4G11 and D2, have sequence homology with TSH α and β subunits. Peptides from the CDRs derived from these monoclonals inhibited antibody binding to and TSH cAMP production of FRTL-5 rat thyroid cells [128]. Synthetic peptides representing a single CDR reproduced the antagonistic physiology of the mAb. The peptide may mimic the physico-chemical topography of the antibody; or functionality can also be imparted through adaptations in the recipient molecule or orientation of residues of the peptide to permit appropriate bond formation.

### 2.5. CDR Based Viral Inhibitors

#### 2.5.1. Anti-Reovirus Mimetics

The first report of successful development of a small peptide mimetic that antagonize reovirus binding to its receptor was designed from the CDR loops of an antibody. The anti-reovirus inhibitor design was based on the observation that the anti-idiotype antibody can mirror the nature of antigen led to the discovery of small synthetic molecules to [40,44,132]. The peptidomimetic was developed from the Ab2 (87.92.6) which manifested an internal image of reovirus hemagglutinin type 3 (HA3) [44,133]. MAb 87.92.6 was raised against a reovirus neutralizing mAb, 9BG5 [134], and found to serve as an anti-reovirus type 3 receptor (Reo3R) antibody. MAb 87.92.6 binding to lymphoma and neuronal cell surfaces competitively inhibited reovirus binding [135,136].

Significant sequence homology between the second CDRs of the light and heavy chains (L2 and H2, respectively) of mAb 87.92.6 was observed with HA3 [40], and synthetic peptides corresponding to these CDRs were tested for their ability to mimic mAb 87.92.6. The linear L2 peptide at high concentrations inhibited both mAb 87.92.6 and reovirus binding to cells [130] and inhibited ConA-induced proliferation of lymphocytes [133]. H2, while playing a minor role in 9BG5-87.92.6 interaction, does not mediate 87.92.6 binding of Reo3R [130]. Furthermore, immunization with the L2 peptide coupled to a carrier effectively elicited an anti-reovirus 3 neutralizing antibody response, whereas the H2 peptide was ineffective [132].

With the identification of the residues involved in conferring biological activity of mAb 87.92.6 using computer modeling the structure of the mAb 87.92.6 L2 site was determined and compared to the HA3 epitope. Both the mAb 87.92.6 L2 and the HA3 region of sequence homology were predicted to be structures that are reverse turn loops. More specifically, the L2 site was predicted to be a distorted β hairpin loop, while the HA3 epitope represented a type 1 β-turn with a G1 β bulge [44]. This peptide mimetic was then developed into a non-peptidic form using synthetic chemistry [3] revealing the feasibility that CDRs alone can initiate biological effects similar to an antibody. These anti-reovirus antibody mimetics were not optimized as inhibitors but the creation of a synthetic antibody loop was an important step in creating new types of therapeutics.

#### 2.5.2. Structure-Based Design of Antibody-like Mimetics

Work primarily from Roberto Poljak [58,137] revealed that CDR loops are the functional units of an antibody’s binding features. Recently, Nakajima *et al*. [138] have developed a new structure-base approach to develop the mimics of pertuzumab based p185^erbB2/neu^-antibody complex using the epitope and paratope information from the antibody-Her2 complex, computationally estimated amino acid positional fitness (APF) in their design, and docking studies to develop a small tetra-peptide, Ac-Pro-His-Ala-His-Phe [138]. The peptide, HRAP enhanced paclitaxel-induced apoptosis of breast cancer cells in a combined treatment approach [139].

**Figure 2:**
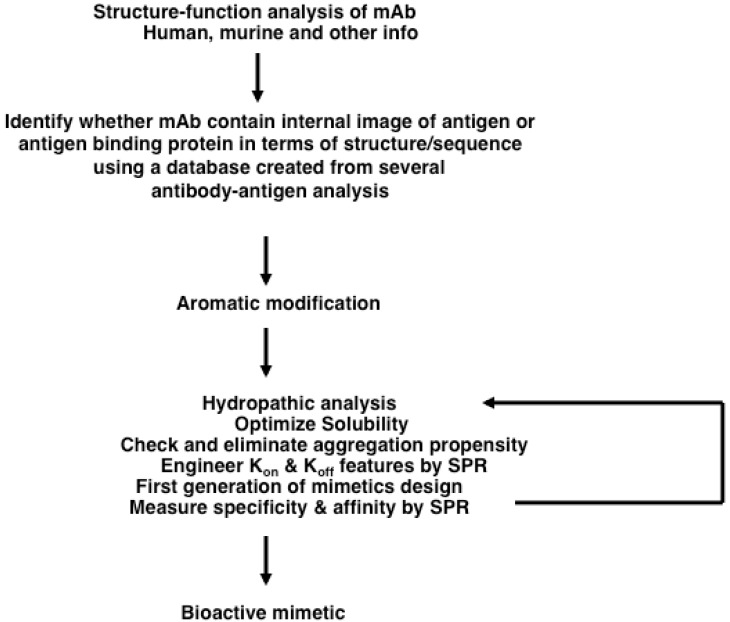
Overall scheme in designing the antibody mimics. A systematic approach has been developed to design antibody mimic based on the three-dimensional structure of Fab, which involve limited bioinformatics, computational biology and use of surface plasmon resonance (SPR).

We have created a structural-database from the analysis of about 50 antigen-antibody complexes in the Protein Data Bank [55,140]. The database consist of primary sequences of CDR regions, definition of conformational features of CDR loops in terms of dihedral angles (φ, ψ), contact residues types, and solvents if any. Combining the observation that some antigens/antibodies mirror some aspects of conformational complementarity, and the variation in the results from the database, a streamlined approach has been developed (Figure 2). Some aspects of the approach are discussed in our efforts to develop anti-p185^erbB2/neu^ (also known as p185^Her2/neu^ or Her2/neu or Her2) and anti-EGFR mimetics.

### 2.6. CDR Based p185^erbB2/neu^ Receptor Inhibitors

The human homologue of *neu*, *c-erbB-2*, Her2 was identified and characterized [94,141,142]. The oncogenic point mutation found in p185^neu^ has not been found associated with human neoplasia, but the human p185^erbB2/neu^ protein is overexpressed in a variety of adenocarcinomas typically as a result of erbB2/neu gene amplification. A number of studies have suggested that overexpression of erbBr2/neu is closely linked to the neoplastic process. Observation of p185^erbB2/neu^ amplification was first described for a human gastric tumor [143,144,145] and Slamon and colleagues [146,147] examined the protein, DNA, and RNA levels of *c-erbB-2* in breast and ovarian adenocarcinomas and correlated p185^erbB2/neu^ amplification with a poor clinical outcome. Amplification of the erbB2/neu gene and subsequent overexpression of p185^erbB2/neu^ was identified in 25%–30% of primary breast and ovarian tumors. Tumors with higher gene copy numbers of erbB2/neu correlated with a poorer patient prognosis. Some, but not all, studies have confirmed these results and have been the subject of several reviews [148,149]. P185^erbB2/neu^ overexpression also appears to be associated with non-small cell lung [150], stomach and colon [151], and a high percentage of pancreatic adenocarcinomas [152]. The importance of levels of the p185^erbB2/neu^ protein as a prognostic indicator is supported by studies demonstrating a functional linkage between p185^erbB2/neu^ overexpression and cellular transformation. Studies by Drebin *et al*. [153,154,155] and DiFiore and others (reviewed in [156]) describes *in vitro* and *in vivo* assays correlating the overexpression of human p185^erbB2/neu^ or rat p185*^neu^* with their transforming activity.

Our original work led to the discovery of several anti rat p185^erbB2/neu^ monoclonal antibodies that were biologically active and able to reverse the malignant transformation of neu oncogene transformed cells [154]. The prototypic antibody 7.16.4 was able to bind both rat and human forms of neu proteins. Later monoclonal antibodies were developed using similar immunization strategies but employing human proteins and cells as immunogens. This led to the development of the 4D5 monoclonals [157]. These 4D5 antibodies were humanized [158] leading to rhuMAb 4D5 (trastuzumab) [153,154,155,157,159,160]. Trastuzumab (Herceptin) is now widely used in the treatment of breast cancer.

#### 2.6.1. Comparison of Rat and Human Forms of Monoclonal Antibodies

**Figure 3:**
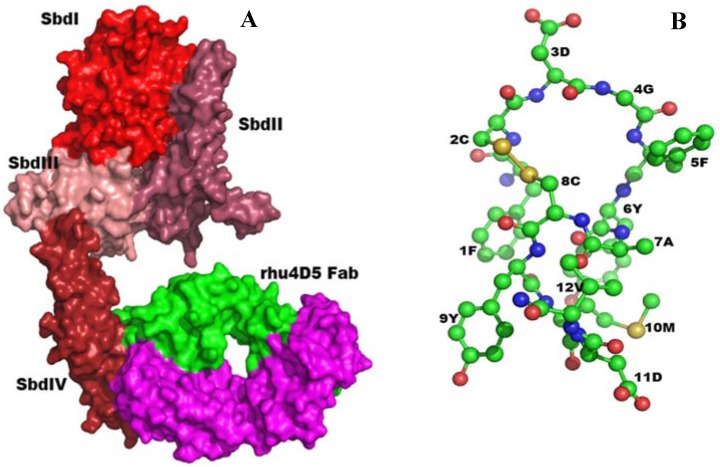
(**A**) Three-dimensional structure of p185^erbB2/neu^ complexed with rhu4D5 (Herceptin) (PBD code: 1N8Z [165]) is shown in molecular surface model. The ectodomain of Her2/neu consists of four subdomain, and each are shown in different shades of red color. The antibody, rhu4D5 binds to the membrane proximal, subdomain IV (SbdIV) of Her2. Based on the shared binding features, the mAb 7.16.4 is also expected to bind to the membrane proximal SbdIV. (**B**) Solution structure of anti-Her2/neu peptidomimetic (AHNP) as determined by NMR is shown in ball-and-stick model. Atoms are colored as follows: Carbon (green), Nitrogen (blue), Oxygen (red), and sulfur (yellow). Amino acid residues in AHNP are indicated by one letter codes.

Two anti-p185 antibodies: the monoclonal antibody 7.16.4 and rhuMAb 4D5 which were raised against the the ectodomain of rat (neu) and the human p185^erbB2/neu^ homologue respectively showed that the structure of these two antibodies are structurally similar in the variable region, especially the CDR3 region, which dominantly determines antibody-antigen interactions [161]. Functionally 7.16.4 can also inhibit p185^erbB2/neu^ mediated proliferation and transformation. Furthermore 7.16.4 compete with trastuzumab (Herceptin) for binding to cell surface of p185^erbB2/neu^ [161]. Reciprocally, the rhuMAb 4D5 shows binding to the rat p185neu indicating that these two antibodies share an epitope on the p185 receptor [161] (Figure 3A). These observations suggested that the conformation adopted by HCDR3, which as mentioned is the most dominant binding surface of the antibody, might be crucial for the binding to the ectodomain of p185^erbB2/neu^ receptor.

The anti-p185^erbB2/neu^ antibody mimetic—The initial attempts to create a mimetic consisted of several peptide analogues derived from L1 and H3 of both 7.16.4 and 4D5 [162]. Since the two antibodies share an overlapping epitope, the secondary structures adopted by the CDR peptide analogs were expected to adopt similar backbone conformations in the complex even though they slightly differ in primary sequence. Structural comparison of the trial structures was then used to screen for potential candidate. One of the exocyclic peptidomimetics (Phe-Cys-Gly-Asp-Gly-Phe-Tyr-Ala-Cys-Tyr-Met-Asp-Val) showed moderate activity. A careful analysis by high-performance liquid chromatography (HPLC) revealed two prominent peaks and isolation of the peaks showed that one species had higher activity. Upon sequencing, it was noted that the glycine3 was missing. A model without glycine3 revealed a more rigid and preference to adopt a classical β-turn than the original peptide. The putative contact residues of the mimetic appear to have a comparable relative disposition [root-mean-square (rms) deviation for Cα atoms is 2.2 Å] to that of the parent antibody [48]. To enhance stability folding and avidity, aromatic modification at the termini was employed [42,163,164]. In addition to amino acid residues from CDR, amino acid residues proximal to the CDR may be involved in antigen interaction [56]. We have used extended residues (Met-Asp-Val) beyond the stabilizing cysteine residues for all of the Anti-Her2/Neu Peptidomimetic (AHNP) species we created. The Met-Asp-Val residues were chosen from the framework region of the 4D5 antibody to extend the surface area at the interface of interaction. The solution structure of AHNP is shown in Figure 3B.

The entropy loss in the conformation of the peptidomimetic (deletion of flexible glycine) resulted in 300 nM binding affinity for the ectodomain of p185^erbB2/neu^. Thus, exocyclic peptides that adopt rigid and comparable ring sizes to β-turns may be expected to show high affinity and binding activity. Though the K_d_ of the AHNP is less potent than that of the monoclonal antibody, their K_off_ rate is comparable which suggests that both antibody and AHNP form a stable complex.

#### 2.6.2. *In Vivo* Tumor Growth Inhibition by AHNP

Shepard *et al*. [166] have shown that murine monoclonal antibody 4D5 localizes to the site of tumors in athymic mice and inhibits the growth of p185^erbB2/neu^ overexpressing human tumor xenografts. We demonstrated that AHNP mimic the antibodies’ function *in vivo* [48].

*In vivo* growth of T6-17 transfected fibroblasts expressing human p185^erbB2/neu^ was evaluated in athymic mice. We have tested the efficacy of AHNP in two set of studies: (1) AHNP was administered intraperitoneally (IP) three times weekly following inoculation of tumor cells in the flank. Sustained treatment with AHNP resulted in inhibition of tumor xenograft formation (*i.e*., prevent tumor formation) and (2) When AHNP administered intraperitoneally (IP) after the development of small palpable tumors derived from the T6-17 fibroblast, AHNP inhibited progression of tumor formation in these animals. These observations show that AHNP could inhibit progression of growth of established tumors.

**Figure 4:**
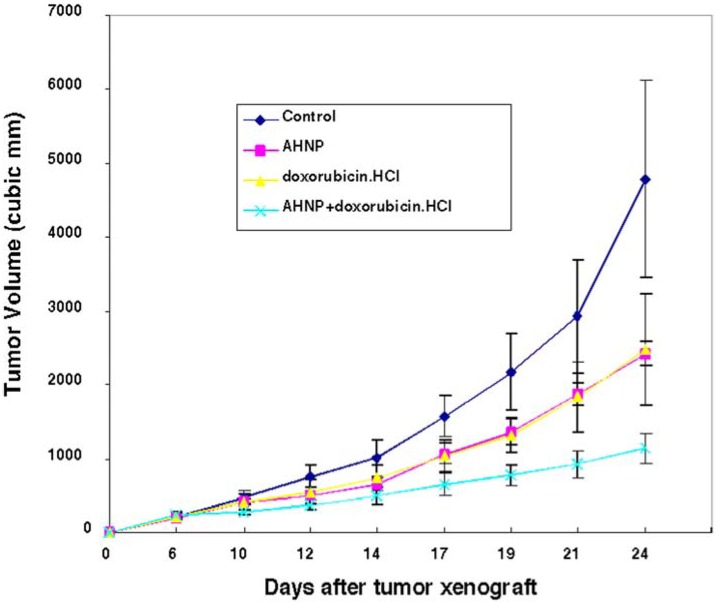
Inhibition of tumor growth in mice by AHNP: About 2 × 10^6^ T6-17 cells suspended in 200 μL of PBS were injected subdermally in the right thigh of nude mice. Six days after tumor allograft, tumors reached approximately 200–230 mm^3^ in volume. Animals were regrouped into 4 treatments groups-control, AHNP alone, doxorubicin alone, and AHNP in combination with doxorubicin. 100 μg of doxorubicin was given at day 6 and day 20. AHNP was administered (200 μg) intraperitoneally three times a week from day 6 after tumor allograft. Tumor growth was monitored three times weekly for 4 weeks. Tumor volume was calculated by the formula: π/6 × (larger diameter) × (smaller diameter)^2^. The figure is reproduced from Park *et al*. [48] with permission from Nature.

Since AHNP showed increased apoptosis of tumor cells when treated with chemotherapeutic agents *in vitro*, we examined the same effects *in vivo*. We investigated the effects of AHNP treatment combined with doxorubicin *in vivo*. We compared the *in vivo* growth of already-established T6-17 tumor xenografts in athymic mice. Although AHNP and doxorubicin independently showed inhibition of established tumor growth, administration of both AHNP and doxorubicin additively increased growth inhibition of tumor xenografts (Figure 4).

Thus AHNP demonstrated remarkable biological activity and high affinity for the p185erbB2/neu receptor. These studies indicate that the approach described here can be translated into clinical use after further modification of AHNP to improve its pharmacokinetics.

Trastuzumab (Herceptin) mediates its tumor effects by receptor downmodulation, and ADCC as well as through cell cycle arrest [167,168]. AHNP cannot engage ADCC mechanisms, but is potent *in vivo*. Based on the interface mimetics derived from p185^erbB2/neu^ [169], we hypothesize that AHNP by binding to the membrane proximal domain of p185^erbB2/neu^ promote a defective complex concomitantly altering the p185^erbB2/neu^ mediated signaling pathway. We are currently investigating if the mimetics promote defective receptor complex using X-ray crystallography.

### 2.7. AHNP Function Is Context Independent in Terms of Adjacent Peptidic Regions

The constrained exocyclic peptide AHNP functionally mimics intact antibody albeit with a lower affinity. Generally attempts to use CDR grafting and CDR based affinity maturation are restricted to immunoglobulin fold containing proteins. It has been unclear if the structure and function of CDR peptidomimetics such as AHNP would be retained in the context of non-immunoglobulin proteins. This feature is of importance if AHNP is to be exploited for diagnostics and therapeutics.

To investigate this property of environmental context, we fused the AHNP peptidomimetic to several non-immunoglobulin proteins such as streptavidin, IP-10/CXCL10 and vimentin. AHNP fused to tetrameric streptavidin (SA) revealed increased avidity (8.8 nM) and retained significant comparable biological activity to the h4D5 [170]. These studies suggest that peptidomimetics derived from the CDR of antibody can function in a context independent manner. Zhang *et al*. [171] have found the AHNP fused SA has been successfully to bind p185^erbB2/neu^ proteins with high affinity.

The implication of context independence of CDR like loops is that it represents an intrinsic feature of exocyclics such they can operate in any framework and this feature anticipated their use in so called humanization of antibodies [172,173].

### 2.8. AHNP Is a Novel Small Molecular Probe for p185^erbB2/neu^ Biology, Diagnosis, Drug Delivery and Therapeutics

One of the advantages of reducing the mass of the targeting agent is an increased diffusional penetration into the tumor. For example, it has been estimated that a 150-kDa molecule would require one week to reach an intratumoral concentration equal to one-half its concentration in the blood at a distance of 1 mm from the vessel wall while a 400 Da molecule would require less than an hour [174]. The AHNP molecule is about 1.5 kDa which is still larger than a typical small molecule, but it can be used as a template to design a much smaller non-peptidic molecule from its three dimensional structure as reported in the case of reo virus inhibitors [3]. On the other hand, it is much smaller than soluble Fv, which are being developed for cancer treatment [72,73,93]. Fantin *et al*. [175] engineered a chimeric peptide, BHAP by conjugating AHNP to a mitochondrial proapoptotic peptide, PAP to target Her2 expressing tumor cells. The chimeric peptide selectively targeted Her2 expressing human breast cancer cells including Herceptin resistance cell lines, and inhibited tumor growth *in vitro* and *in vivo*. Furthermore, Fentin *et al*. [175] conjugated BHAP to biotin to create a tetrameric unit and targeted Her2 expressing human breast cancer cell lines. The tetrameric BHAP show significant (80 fold improvement over BHAP) biological effect in breast cancer tumor cells. These studies show that as a small peptide AHNP is facile in developing new therapeutics.

*In vitro* and *in vivo* evaluation, AHNP functions like the monoclonal antibody, Trastuzumab (Herceptin). This general procedure do not require humanization which is a laborious process and yet can elicit human anti-mouse antibody (HAMA) [27,83,90,176]. AHNP is a better candidate for tumor treatment in this regard. In preliminary experiments, AHNP shows a reasonable *in vivo* stability. AHNP can be improved for better pharmacokinetics. Since AHNP mediated enhanced growth inhibition when combined with chemotherapeutic agents such as doxorubicin, radiation [48] and taxol [177], AHNP may serve a role in the treatment of tumors. Use of Trastuzumab in combination with anthracyclines in breast cancer treatment displayed cardiovascular toxicity [178]. Currently, no reliable tests available to predict cardiotoxicity of p185^erbB2/neu^ targeting agents. Nonetheless, in a preliminary study, AHNP has been shown to less effect on cultured atrial myocardial tissues compared to the anti-Her2 antibody [179] treatment suggesting that targeting p185^erbB2/neu^ receptors using a small molecule might obviate the side-effects of antibody based therapies.

Small antibody fragments have been engineered for radioimmunotherapies [176], but their success is limited by the large size and circulating half-life [14,72,73]. The smaller size of AHNP and its high affinity binding to ectodomain makes it a suitable candidate for immunotherapy and as a diagnostic agent. Towards this goal, we attempted improved the affinity and half-life of AHNP by fusing the peptide to streptavidin (SA).

AHNP as vector for drug delivery—Recent advances in the development of nanoparticles opened up a new avenue for diagnostics and drug-delivery. Earlier, we shown that bi-functional conjugated with taxol as effective approach for tumor specific drug delivery [177]. To expand the potential of AHNP, we developed AHNP based polymersome based nanoparticles for delivery of doxorubicin [180]. In a preliminary study, AHNP conjugated forms showed moderate efficacy in delivering the chemotherapeutic agent to tumors in mice [181]. One of the reasons for the sub-optimal activity was traced back to AHNP conjugation to the polymersomes, where aromatic residues included for the stability in AHNP promoted aggregation in the presence of polymer. Furthermore, the terminal aromatic residues collapsed the conformation due to clustering of hydrophobic residues based on computer simulation studies [140]. Currently, AHNP is being reengineered so that it can be used in poly-β-maleic acid based nanoparticles.

While AHNP need to be reengineered for nanoparticles, some novel uses of AHNP have been reported. Afshar *et al*. [182] have fused AHNP to the *C*-terminus of mutant human purine nucleoside phosphorylase to deliver prodrugs that upon delivery induce cytotoxic effect to tumor cells. Other applications include fusing streptavidin (for detection/diagnosis purposes) [170] and using cell-penetrating TAT for targeting transcription factor involved in p185^erbB2/neu^ transduction [183].

### 2.9. CDR Based Epidermal Growth Factor Receptor (EGFR) Inhibitors

We have had reasonable success in creating anti-erbB mimetics that disable p185^erbB2/neu ^*in vitro* and *in vivo* [48]*.* We therefore used the deduced structure of the monoclonal antibodies C225 and 425 resolved by X-ray crystallography [184] to design anti-EGFr mimetics. A bioactive anti EGF receptor Peptidomimetic, (AERP) has been designed using the C225 heavy chain CDR3 region as a template.

**Figure 5:**
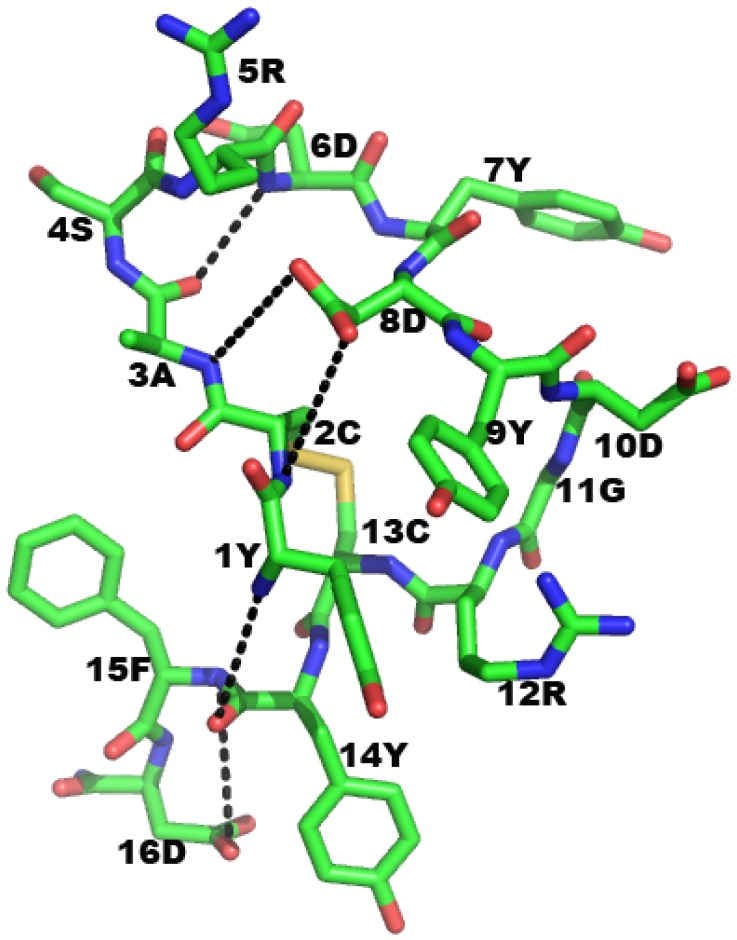
Three-dimensional structure of AERP. Molecular model of AERP (carbon-green; nitrogen-blue; oxygen-red and sulfur-yellow) is in stick model. The exocyclic peptide, AERP adopt a combination of a β-turn and an anti-parallel β-sheet structure to create a large ring-size structure. Amino acid residues are indicated in one letter code. Intra-molecular hydrogen bonds are shown in dash-lines.

It appears to adopt a β-turn and an anti-parallel β-sheet secondary structure as deduced through minimization modeling studies (Figure 5). Conformational features combined with the pharmacophore from the CDR3 loop appear to be responsible for the bioactivity we have observed. These mimetics are modified to include aromatic amino acid residues to enhance the stability of folding and avidity [42]. In addition, we have incorporated a small tail of three framework-derived residues, which we have found to provide additional functional surfaces for binding [48].

We obtained baculovirus-produced and partially purified ectodomain forms of EGFr species from Lemmon (University of Pennsylvania). The ectodomain construct was further purified by high performance gel filtration chromatography.

Kinetic binding characteristics of AERP to the ectodomain of the EGF receptor were studied using biosensor techniques showed that AERP binds to the EGF receptor with an approximate affinity of 400 nM. At optimum surface density (3600 RU), AERP bound to EGF receptors in a concentration-dependent manner with a dissociation pattern (k_off_) within an order of magnitude to that of the C225 mAb (data not shown). In a preliminary study, AERP inhibited EGF mediated tumor growth in transformed cell lines. In NE99 cell lines that overexpress the EGFR [185], treatment with the AERP resulted in a dose dependent 40% inhibition of cell growth driven by recombinant EGF (data not shown). On the other hand, Jurkat cells which do not overexpress EGF receptors were unaffected by AERP or CD4.M3 (an unrelated anti-CD4 mimetic) treatment (data not shown). Unexpectedly, AERP inhibited cell growth of EGFR and p185^erbB2/neu^-expressing cells suggesting that AERP might bind to epitope shared by EGFR and p185^erbB2/neu^. Interestingly, when AERP and AHNP where synthesized as chimera, the anti-tumor activity in transformed cells were comparable to anti-p185^erbB2/neu^ antibody (Table 1). Further improvement of the chimera is being developed for diagnostics and therapy.

**Table 1:** Anti-Proliferative effects of AERP-AHNP chimeric peptide.

Peptide Mimetic	Concentration (μg/mL)	% Inhibition	
		T6-17 (Her2++)	A431 (EGFR++)
AHNP	10.0	73.92	19.07
	1.0	48.03	22.63
AERP	10.0	10.08	15.44
	1.0	0.35	1.63
AERP-AHNP	10.0	92.82	22.51
	1.0	72.25	11.86
h4D5 (trastuzumab)	1.0	51.07	8.77

### 2.10. AERP as Single-Photon Emission Computed Tomography (SPECT)-Agent for Tumor Imaging

AERP binds to both p185^erbB2/neu^ and EGFR with reasonable affinity, and thus potentially a suitable candidate for diagnosis purposes. In a preliminary study, AERP was coupled to SPECT agent ^99m^Tc and used for tumor imaging in breast cancer animal model. AERP coupled ^99m^Tc through diethylene triamine pentaacetic acid (DTPA) showed tumor-specific accumulation. The tumor-to-blood ration was 3.2 comparable to that of scFv [49]. However, the conjugated peptide also retained significant amount in liver and kidney. Further work is in progress to improve peptides’ pharmacokinetics for diagnosis of breast cancer.

## 3. Future Direction for Peptides as Therapeutic Agent

Peptides that mimic antibodies are novel species with great potential for diagnosis, and treatment, which remains to be validated. However, peptides’ short half-life and weak affinity compared to antibody are main obstacles for the peptides to be useful in clinical settings. It remains to be examined if lack of ADCC capabilities by the antibody mimic will also limit its utility. To overcome some of the limitation, we have attempted to engineer antibody mimics for targeted delivery. Since our work demonstrated that a large antibody can be reduced to a small peptide, several studies report modified peptides either chemically to as fusion proteins hold greater promise for therapeutic purposes. A great need is the engineering of these peptides as oral drug, which would not only enhance the drug pipeline, but also would greatly reduce healthcare costs.

## 4. Conclusions

We have successfully developed a method to reduce the macromolecular structure of a monoclonal antibody to a small secondary structure mimetic that has high affinity and *in vivo* activity against tumor growth. AHNP functionally mimic the antibody function and thus it can be considered as a true “antibody mimic”. As mentioned before, creation of such small antibody mimics not only eliminates the laborious humanization of antibodies, but also provides a new avenue in the design of antibody-based therapy. To our knowledge, AHNP and AERP are the first rationally designed anti-receptor small peptidomimetics that bind to the ectodomain of an oncoprotein. We believe that this approach may lead to the design of small molecule compounds which may be used as novel receptor-based anticancer therapeutics in man.
